# From Aesthetic to Functional Dilemma: Managing a Pedunculated Giant Basal Cell Carcinoma of the Nose

**DOI:** 10.7759/cureus.81175

**Published:** 2025-03-25

**Authors:** Vasileios Psarras, Markos Antonopoulos, Balasis B Stavros, Dimitrios Kehagias, Georgia Kyriakou

**Affiliations:** 1 Department of Surgery, University General Hospital of Patras, Patras, GRC; 2 Department of Emergency, University General Hospital of Patras, Patras, GRC; 3 Department of Plastic Surgery, Patras University Hospital, Patras, GRC; 4 Department of Dermatology, University General Hospital of Patras, Patras, GRC

**Keywords:** basal cell carcinoma, functional status, nose diseases, oncology, plastic surgery

## Abstract

Giant basal cell carcinoma (GBCC) is a rare and aggressive variant characterized by its frequent invasion of underlying tissues and the occasional development of metastases. An even rarer form, pedunculated GBCC, can lead to functional complications depending on its location. This report presents a case of pedunculated GBCC located on the nose of a 93-year-old patient, which resulted in feeding intolerance. After an R0 surgical resection, histopathological examination confirmed the adenoid type of basal cell carcinoma, with no evidence of distant disease. In the literature, only one case of pedunculated GBCC in the facial region has been documented. A multidisciplinary approach involving prompt investigation and treatment is essential to prevent functional issues that could compromise the patient's quality of life.

## Introduction

Polypoid basal cell carcinoma (BCC) is a rare and clinically distinct subtype of BCC. It is characterized by a pedunculated, stalk-like connection to the skin surface and histopathological features showing tumor aggregates confined to the exophytic polypoid area [1]. Although typically slow-growing, this tumor can become more aggressive, potentially developing into the exceedingly rare giant basal cell carcinoma (GBCC). Beyond its malignant potential, this variant can significantly impact a patient's quality of life, particularly depending on its location, necessitating prompt management and a multidisciplinary treatment approach. The aim of this case report is to present a quite rare occasion of a pedunculated GBCC that severely affected the patient's quality of life.

## Case presentation

**Figure 1:**
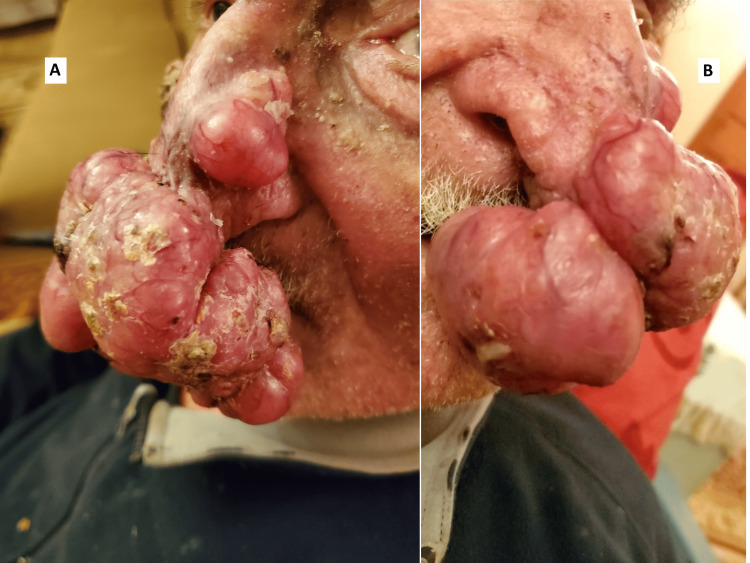
(A) Left side and (B) right lateral side of the giant pedunculated tumor hanging from the patient's nose which was hindering oral food intake.

For the presentation of the case report, an informed consent of the patient was acquired. A 93-year-old Caucasian man was referred to our department due to a three-year history of two progressively enlarging, non-tender, exophytic, pedunculated nodules hanging from his nose. Physical examination revealed a large skin-colored nodule (8.3×7.5×4 cm) with yellow crusts on the tip of the nose and a second, smaller nodule (3.5×1.5×1.5 cm) on the left lateral side. Both lesions had a pearly, smooth surface and were marked by telangiectatic vessels at the periphery (Figure 1). Neck palpation did not reveal any regional lymphadenopathy.

The patient's chief complaint centered on the significant impact these nodules had on his functional and psychological well-being, severely diminishing his quality of life. Functionally, the nodules interfered with his eyesight and ability to eat, since he often had to hold the nodule with his left hand to bring food to his mouth. This led to considerable weight loss and frailty over the past two years. Other potential causes of unintentional weight loss were ruled out by the attending physician. Psychologically, the presence of the lesions caused anxiety and distress, leading him to avoid family and social gatherings over the past year, which further diminished his cognitive reserves and increased his vulnerability. His medical history included prostate cancer, successfully treated with radical prostatectomy 20 years ago, and chronic obstructive pulmonary disease, managed with anhydrous ipratropium bromide.

The clinical differential diagnosis primarily included BCC, but other potential neoplasms such as lipofibroma, fibroepithelioma of Pinkus, amelanotic melanoma, squamous cell carcinoma, and Merkel cell carcinoma were also considered. A dermoscopic evaluation revealed typical arborizing vessels and foci of blue-gray ovoid nests. Based on these clinical and dermoscopic findings, BCC was highly suspected. Imaging studies were not performed due to the inability of the patient to stand for a magnetic resonance imaging (MRI). Also, due to his 93-year-old age, any imaging would not change the surgical plan, since removal of this lesion for functional reasons was mandatory.

Given the patient's reluctance to undergo general anesthesia or receive vismodegib, an FDA-approved medication for BCC, we opted for surgical excision under local anesthesia. Due to the lesions' high vascularity, a combined approach was employed using bipolar diathermy and suture ligation for larger vessels. We first excised the larger, more pedunculated nodule and then extended the incision to remove the second nodule. Careful hemostasis was maintained throughout the procedure to avoid trauma to the nasal cartilage. A full-thickness skin graft was harvested from the right subclavicular area and sutured over the nasal defect with interrupted Vicryl 4-0 sutures. The procedure, conducted in the surgical theater, lasted approximately 30 minutes.

**Figure 2:**
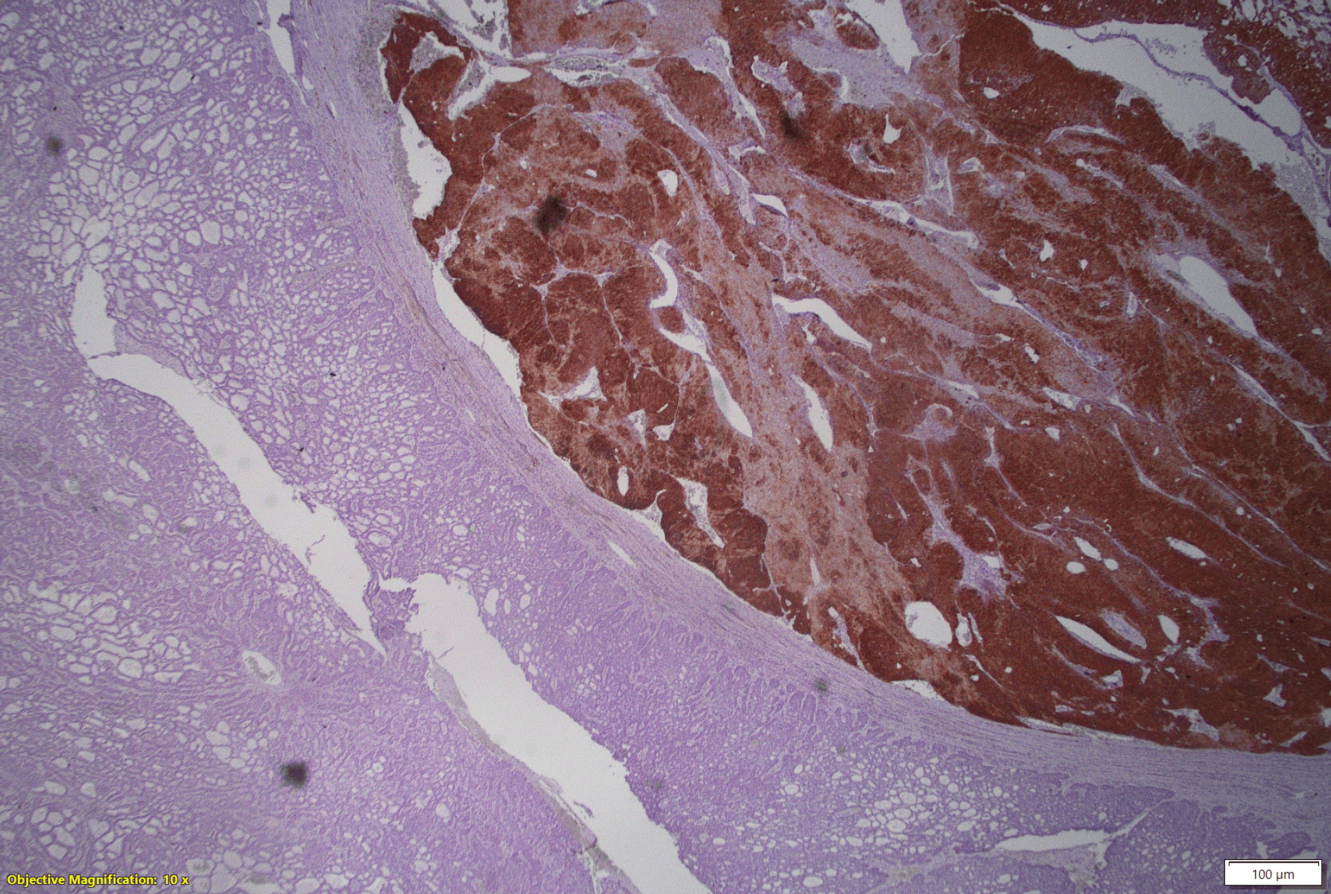
Microphotograph showing adenoid pattern with arrangement of the lesional tissue in a lobular pattern (H&E, 100×). The diagnosis was confirmed with the use of BerEp4 stain. H&E: hematoxylin and eosin

Histopathological examination of both specimens confirmed the adenoid type of metatypical BCC, with tumors extending into the hypodermis (Figure 2). Surgical margins were clear, indicating an R0 resection.

**Figure 3:**
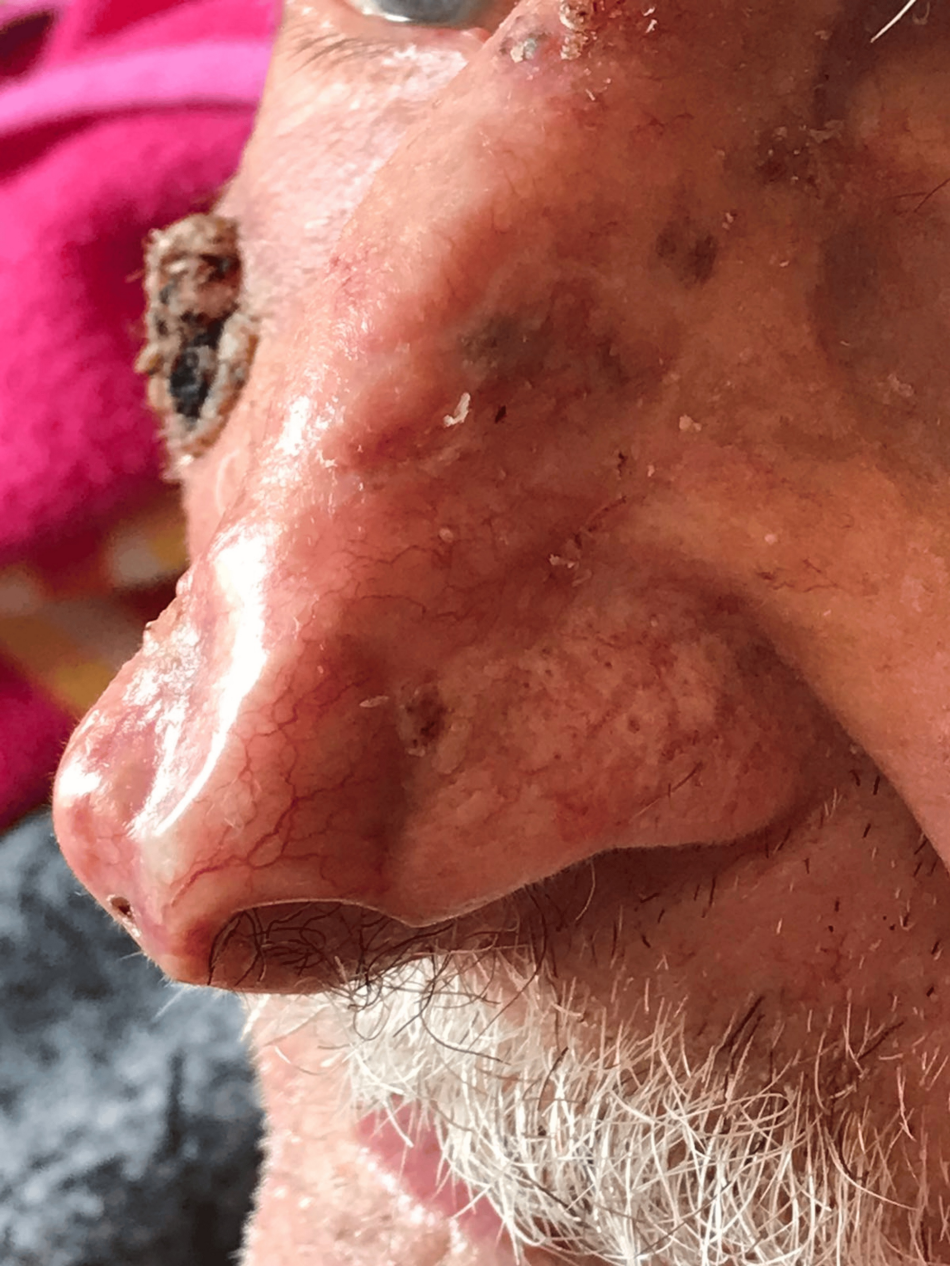
An excellent cosmetic and functional result was achieved eight weeks after the operation. The patient was submitted to excision of a third tumor, observed over his right cheek, a few weeks later.

The postoperative course was uneventful, and at his last follow-up visit, eight weeks post-surgery, complete healing with minimal scarring was observed (Figure 3). The functional and psychological outcomes were remarkable, since the patient could return to his usual activities and eat without difficulty, significantly enhancing his quality of life. He expressed satisfaction with both the cosmetic outcome and the overall improvement in his well-being.

## Discussion

Pedunculated BCC is a rare variant characterized by its polypoid appearance and a pedicle that attaches it to the skin. It is characterized by an aggressive biological behavior, with extensive local invasion, frequent metastasis, and associated poor prognosis. An even rarer variant is GBCC, which accounts for 0.5-1% of BCCs and is defined by a diameter greater than 5 cm. There are limited cases of pedunculated BCCs documented in the literature, while there is only one reported case of a pedunculated GBCC in the facial region [1-4]. GBCCs exhibit aggressive biological behavior, frequently invading underlying tissues and occasionally leading to metastasis [5]. While metastasis is generally rare in BCCs, GBCCs are considered high-risk according to the National Comprehensive Cancer Network (NCCN) Clinical Practice Guidelines [6].

Currently, specific treatment guidelines for GBCCs have not been established [7-9]. Although complete surgical excision provides the best outcomes, it can pose challenges in terms of functional or cosmetic complications, particularly depending on tumor size and location [10]. In our case, the excision was particularly challenging due to the risk of excessive hemorrhage, as the nose is highly vascular. A margin of 5 mm, due to the high-risk features of the GBCC, was selected. The procedural difficulty was further heightened by the patient's refusal of general anesthesia. Nonetheless, given the functional impairments and feeding intolerance caused by the pedunculated GBCC, surgical excision emerged as the optimal approach, leading to an immediate improvement in the patient's quality of life.

## Conclusions

This case illustrates that giant pedunculated BCC, due to its location, can result not only in aesthetic concerns but also in significant functional impairment, necessitating prompt management from a multidisciplinary team of dermatologists, plastic surgeons, and histopathologists.

## References

[1] Yildiz S, Karaarslan I, Yaman B, Ozdemir F (2017). Dermoscopy and reflectance confocal microscopy in pedunculated basal cell carcinoma. Dermatol Pract Concept.

[2] Megahed M (1999). Polypoid basal cell carcinoma: a new clinicopathological variant. Br J Dermatol.

[3] Hirakawa M, Ishikura Y, Futatsuya T, Yamaguchi R, Shimizu A (2022). Polypoid basal cell carcinoma on the nose tip. Case Rep Dermatol Med.

[4] Kouki C, Kammoun N, Sellami K (2022). Slow-growing pedunculated nodule. Clin Case Rep.

[5] Vaca-Aguilera MR, Guevara-Gutiérrez E, Barrientos-García JG, Tlacuilo-Parra A (2019). Giant basal cell carcinoma: clinical-histological characteristics of 115 cases. Int J Dermatol.

[6] Bichakjian CK, Olencki T, Aasi SZ (2016). Basal cell skin cancer, version 1.2016, NCCN Clinical Practice Guidelines in Oncology. J Natl Compr Canc Netw.

[7] Archontaki M, Stavrianos SD, Korkolis DP (2009). Giant basal cell carcinoma: clinicopathological analysis of 51 cases and review of the literature. Anticancer Res.

[8] Betti R, Inselvini E, Moneghini L, Crosti C (1997). Giant basal cell carcinomas: report of four cases and considerations. J Dermatol.

[9] Sahned J, Mohammed Saeed D, Misra S, Thakkar D (2019). Giant ulcerative basal cell carcinoma with local metastasis: a case report and assessment of surgical techniques. Cureus.

[10] Oliveira N, Tchernev G, Kandathil LJ (2021). Giant mushroom-like neglected basal cell carcinoma of the shoulder with spontaneous bleeding: a successful surgical approach. Eur J Case Rep Intern Med.

